# Computer-Assisted Reconstruction and Motion Analysis of the Three-Dimensional Cell

**DOI:** 10.1100/tsw.2003.70

**Published:** 2003-09-03

**Authors:** David R. Soll, Deborah Wessels, Paul J. Heid, Edward Voss

**Affiliations:** Department of Biological Sciences, The University of Iowa, Iowa City, Iowa 52242, USA

**Keywords:** 3D-DIAS, motion analysis, 3D cell reconstruction

## Abstract

Even though several microscopic techniques provide three-dimensional (3D) information on fixed and living cells, the perception persists that cells are two-dimensional (2D). Cells are, in fact, 3D and their behavior, including the extension of pseudopods, includes an important 3D component. Although treating the cell as a 2D entity has proven effective in understanding how cells locomote, and in identifying defects in a variety of mutant and abnormal cells, there are cases in which 3D reconstruction and analysis are essential. Here, we describe advanced computer-assisted 3D reconstruction and motion analysis programs for both individual live, crawling cells and developing embryos. These systems (3D-DIAS, 3D-DIASemb) can be used to reconstruct and motion analyze at short time intervals the nucleus and pseudopodia as well as the entire surface of a single migrating cell, or every cell and nucleus in a developing embryo. Because all images are converted to mathematical representations, a variety of motility and dynamic morphology parameters can be computed that have proven quite valuable in the identification of mutant behaviors. We also describe examples of mutant behaviors in Dictyostelium that were revealed through 3D analysis.

## INTRODUCTION

The development of the microscope by van Leeuwenhoek in the 17th century might be considered the first major technological advance in the field of cell biology. When the first primitive microscopes were employed in the 19th century to observe different cell types, it became clear that cells not only exhibited different shapes, but also contained complex internal architecture. Therefore, it became imperative that the resolution of the microscope be optimized. Higher resolution was achieved through design and increased precision of the optics, leading to the development of the compound microscope[1]. New microscope technologies then emerged, including fluorescence microscopy, phase contrast microscopy, transmission electron microscopy, scanning electron microscopy, and differential interference contrast (DIC)

microscopy[1]. The application of these new technologies revealed new details of cellular architecture. The development of the electron microscope in particular provided us with ultra-high resolution images of fixed preparations, and revealed subcellular architecture that included macromolecular complexes. Each of these technologies provided 2D information on cell architecture, and to varying degrees had the potential for revealing 3D architecture. Even so, several misconceptions related to dimensionality evolved. First, because of the flat image obtained when viewing a cell from above or below employing conventional compound microscopy, the perception evolved that a cell is basically flat and uniformly attached to the glass surface. Descriptions of cell polarity and organization were therefore primarily 2D, i.e., in the x,y-axes. Second, because humans do not have the capacity for "temporal differencing" (i.e., comparing the shape of a cell at two time points), because cell locomotion is relatively slow, and because we normally monitor a translocating cell qualitatively at one focal plane, we tend to assume that migrating cells are not only 2D, but also that they do not undergo major shape changes when translocating along a flat surface. Third, because we usually analyze cell behavior *in vitro* on a flat glass slide or chamber wall, we tend to think of cellular translocation as a 2D process along a flat surface, when in reality cells may crawl through a complex 3D path of tissue or extracellular matrix.

## THE NEED FOR HIGH RESOLUTION, QUANTITATIVE PHENOTYPES

One of the major goals in understanding how cells crawl is to elucidate the roles of individual cytoskeletal, regulatory, and cell surface molecules in cellular shape, polarity, locomotion, and chemotaxis. In analyzing molecular mechanisms that are involved in these processes, one is struck by the extraordinary number of molecules that have so far been implicated. The list grows ever longer when one considers those biological processes, such as chemotaxis, morphogenesis, and metastasis, in which the basic process of cellular locomotion is manipulated by endogenous and exogenous signals. What is so striking is that the deletion of genes encoding key molecules, like myosin II, has not been fatal to the cell, at least under laboratory conditions[2,3]. In the majority of cases, in fact, deletion mutants are still motile. What has begun to emerge from this apparent paradox is a new perception of how cell shape, polarity, and motility are regulated, and the requirements for assessing phenotype. The exact roles of many of these molecules could only be identified by quantifying shape changes and motility of live wild type and mutant cells using computer-assisted methods. If the method for assessing behavior is of low resolution (e.g., if one only assesses whether or not a cell translocates, or measures the single parameter of velocity), cells harboring mutations in genes that fine-tune behavior will be considered normal, i.e., without a mutant phenotype. It is becoming clear that genes that fine-tune changes in shape, polarity, turning, and the dynamics of pseudopod formation represent the majority of molecules involved in cellular behavior. It is, therefore, obvious that the more detailed and quantitative the description is of normal behavior, the greater one's chances are of identifying a mutant phenotype through comparison with wild type or normal cells. This point is best demonstrated in *Dictyostelium discoideum*, the most popular lower eukaryotic model system for investigating the molecular basis of animal cell locomotion and chemotaxis[4,5,6]. Since *Dictyostelium* is haploid and can be cultured as a microorganism, genes can be deleted using one-step strategies[7]. For this reason more motility- and chemotaxis-associated genes have been deleted or mutated in this cell than in any other amoeboid cell. So far, the deletion of genes for every cytoskeletal element but actin, and deletion of a variety of regulatory elements, have not proven lethal and in almost all cases have not resulted in the cessation of cellular translocation. When deletion mutants were first generated in *Dictyostelium* for a variety of actin-binding proteins and the mutants examined qualitatively, it was concluded that because mutant cells still translocated, either the deleted molecules were not involved in cellular translocation or there was functional redundancy, i.e., other molecules took over the functions of the deleted molecules[8]. Both conclusions proved to be inaccurate in the great majority of cases when methods were employed that increased the resolution of phenotype. When computer-assisted methods were developed to obtain high resolution, quantitative descriptions of the dynamic morphology, locomotion, and chemotaxis of a cell, the behavior of each tested mutant proved to be defective in a unique fashion[9]. When the behavioral defects of mutants harboring mutations in different cytoskeletal and regulatory proteins were demonstrated to be similar, one could tentatively group them in common pathways that could be verified by epistatic studies, and when the behavioral defects of mutants were different, one could tentatively group them in independent pathways. The capacity to interpret common and independent functions correlates directly with the resolution of the behavioral phenotype. Hence, it is not good enough to assess the functional role of a molecule in cellular locomotion by qualitatively assessing whether or not a mutant cell can simply translocate or chemotax.

## THE SOLUTION: COMPUTER-ASSISTED RECONSTRUCTION AND MOTION ANALYSIS OF LIVING CELLS

The solution to the articulated problem has been to develop methods that allow one to reconstruct and quantify in great detail the changes in cell shape, architecture, and behavior that accompany cellular translocation. These analytical methods must be computer assisted and applicable to live, crawling cells[10,11]. The first systems developed, and now in use worldwide, were 2D. Recently, these systems have been expanded to reconstruct and motion analyze cells in 3D. Here we will describe both the 2D and 3D motion analyses systems 2D-DIAS and 3D-DIAS[12]. These systems are based on new edge detection, outlining, wrapping, and presentation techniques that facilitate 2D and 3D reconstruction of the dynamic cell at short time intervals as mathematical representations that can then be used to quantify motility and the dynamic morphology of a cell. 3D-DIAS provides, for the first time, a relatively accurate high-resolution description of a translocating cell in 3D, and reveals just how complex cellular translocation really is.

## 2D-DIAS

The first computer-assisted dynamic image analysis systems, DMS[13,14,15], and the second generation system, 2D-DIAS[10,11], are restricted to 2D reconstructions and motion analysis. Videorecordings or video signals of one or more cells imaged by light microscopy (e.g., bright field, dark field, phase contrast, DIC) at one focal plane are digitized into the DIAS data file, image processed, and outlined either automatically or manually. The outlines are then smoothed and converted to β-spline representations. These representations can be generated at time intervals as short as a thirtieth of a second (normal video time) or at a faster rate with high-speed video camera and recorder. The reconstructions (frames) could be examined individually, used to generate centroid and perimeter tracks (Fig. 1A), or used to generate difference pictures in which cell expansion zones are color-coded green and cell contraction zones are color-coded red (Fig. 1B). Since the final images represent mathematical models, they can be used to compute motility parameters based on the changes in the position of the cell centroid (center of area), and dynamic morphology parameters based on changes in cell contour. The approximately 40 parameters computed by 2D-DIAS can be presented in tabular or graphic form (Fig. 1C) at intervals as short as a thirtieth of a second. Any portion of the cell image (e.g., a pseudopodial region) can be windowed and individually reconstructed and motion-analyzed in a manner similar to the entire cell perimeter.

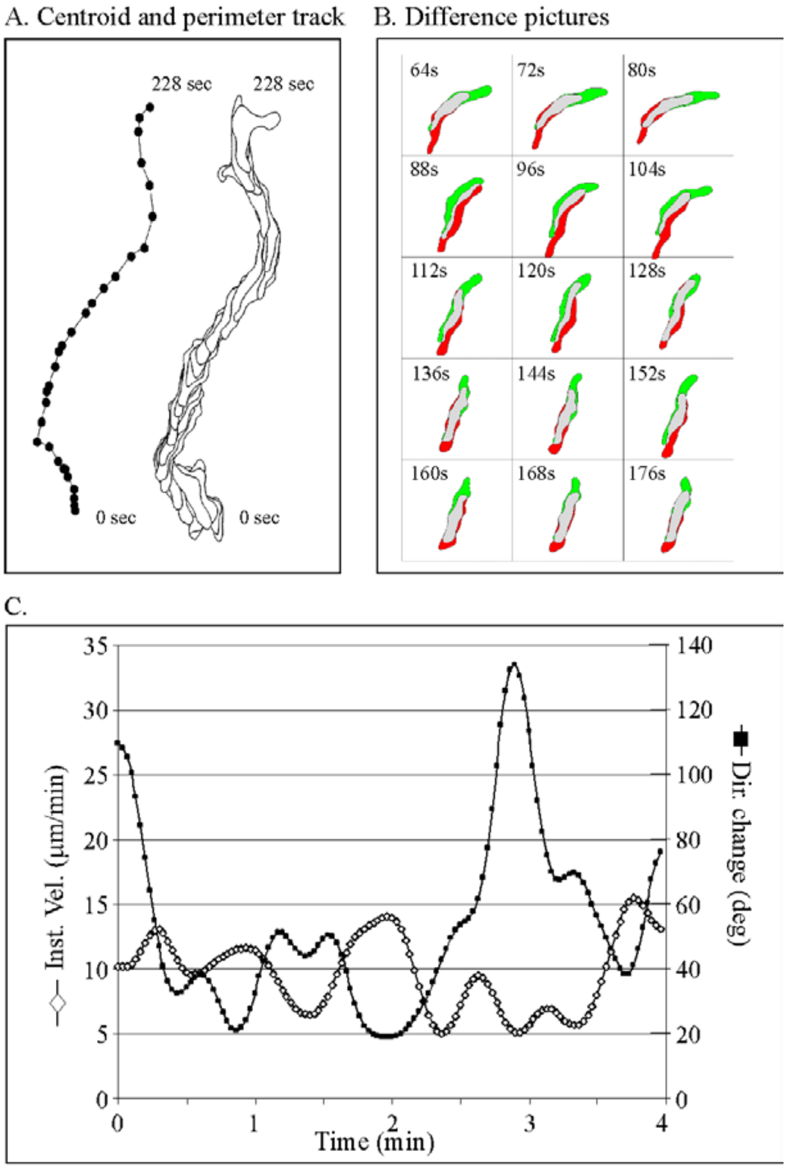

**FIGURE 1.** 2D-DIAS can generate (A) cell centroid and perimeter tracks, (B) difference pictures in which expansion zones are color-coded green and contraction zones are color-coded red, and (C) quantitative data for over 40 parameters, in this case, instantaneous velocity and directional change (see [10] for details). The cell type analyzed is *D. discoideum* strain Ax4 in a spatial gradient of cAMP.

2D-DIAS has proven valuable in describing the basic motility of cells as well as the behavioral responses of cells to spatial gradients, temporal gradients, and natural waves of chemoattractant[16,17,18,19,20,21,22,23,24,25,26]. It has also proven essential in elucidating the behavioral abnormalities of a variety of cytoskeletal and regulatory mutations, and the behavioral characteristics of a variety of normal and abnormal cells in organisms ranging from nematodes to humans[27,28,29,30,31,32]. Because it is 2D, the time necessary to obtain data is much shorter than that for 3D-DIAS analyses, and for that reason, 2D analyses are routinely performed in great detail, while 3D-DIAS analyses are selectively performed. The usefulness of 2D-DIAS, therefore, remains high, and has expanded to the analysis of larvae, heart function, and the behavior of adult organisms[33,34,35]. However, in cases where there is an important 3D component of behavior, and especially in cases when this component is aberrant in a mutant cell line, 3D reconstruction and analysis is necessary. It was, in fact, data obtained from 2D analyses of cell migration that first revealed the need to develop 3D-DIAS. 2D-DIAS analyses of *Dictyostelium* amoebae crawling on a glass surface in the absence of a chemotactic signal (i.e., in buffer) first revealed cyclic changes in cellular area, which suggested either that cells cyclically changed volume or that they underwent cyclic changes in height. By readjusting the focal plane through the z-axis of a moving cell, it became apparent that the latter was the case. It was also a 2D-DIAS analysis of the ponticulin-minus mutant that revealed a slight abnormality in computer-generated cell perimeter tracts that led to the discovery through 3D analysis of unstable pseudopodia moving posteriorly along the dorsal surface of mutant cells[36].

## 3D-DIAS

The 3D dynamic image analysis system (3D-DIAS) began to be developed in the early 1990s (Fig. 2). In this system, optical sections are collected through the z-axis of a cell in a short period of time, and this process is repeated at short time intervals[10,11,12,36,37]. Each optical section in a z-axis collection is outlined, converted to a beta spline representation, stacked, faceted, and smoothed to generate a 3D caged image. A time series of these 3D reconstructions can then be viewed as a dynamic movie through a 3D stereo workstation at any desired angle and any desired speed. Because the outline in each optical section is converted to a β-spline model, the 3D reconstruction represents a 3D mathematical representation, and can, therefore, be used to compute more than 100 motility and dynamic morphology parameters based in the former case on the 3D track of the cell centroid and in the latter case on 3D cell contour changes[10,11].

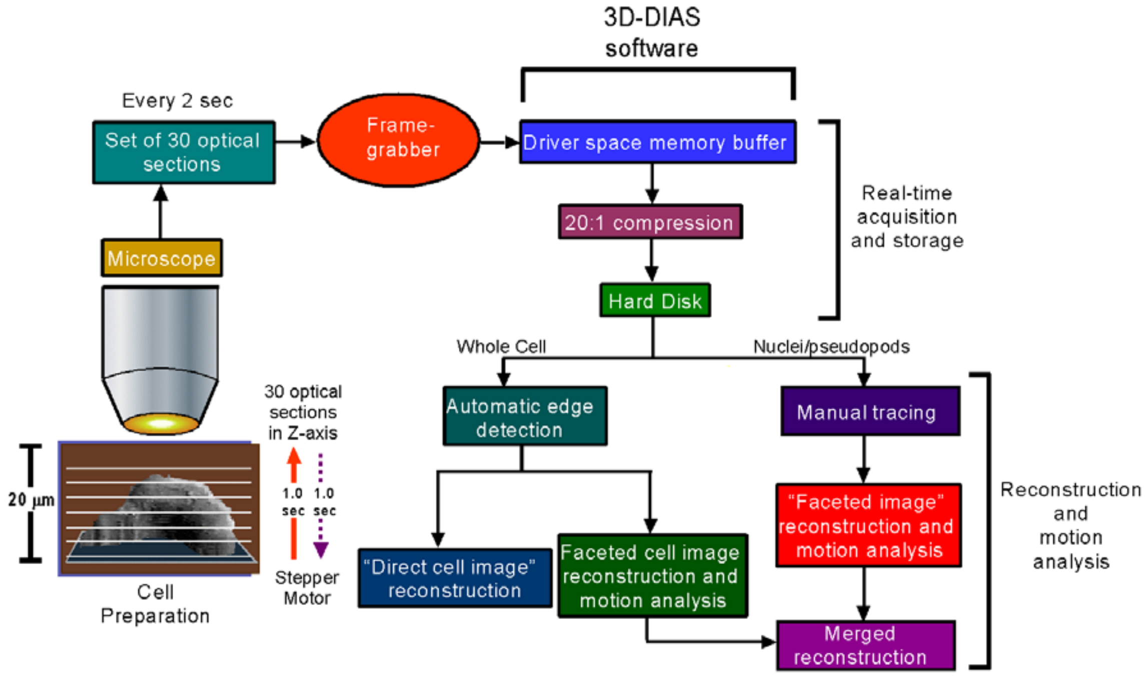

**FIGURE 2**. The major components of "3D-DIAS". A set of 30 optical sections through a live translocating cell is acquired in a 1-s time interval through DIC optics. Optical sections are digitized into a memory buffer and processed as described in the scheme (see [12] for details).

Using a customized computer-regulated stepper motor, 30 optical sections can be collected through DIC optics of a crawling cell in a 1-s period and this process repeated every 2 s (Fig. 2). The short collection time and the slow average speed of *Dictyostelium* or human white blood cell translocation result in no more than a 5% reconstruction artifact due to cell movement through the collection period[36]. The development of a pixel complexity measurement provides automatic outlining of the cell perimeter, even of soft DIC images[37]. Most importantly, because of the use of DIC optics, the perimeters of the nucleus and pseudopodial boundaries can be manually outlined so that 3D reconstructions of nuclei can be inserted into the cell boundaries and 3D reconstructions of pseudopodia demarcated[12]. When combined, one obtains a time series of a crawling cell that includes a transparent, faceted surface (blue), a color-coded nucleus (green), and color-coded pseudopodial regions (red) (Fig. 3). This time series can be viewed in dynamic 3D in a stereo workstation through blue and red glasses in a computer-assisted presentation, or pseudo-3D images can be viewed in a time series in a published figure (Fig. 3). Because nuclei and pseudopodial regions are reconstructed individually in their own trace slots, they can be viewed individually as a function of time and individually motion analyzed[12]. In Fig. 4A, only the nucleus of a living, crawling cell has been reconstructed at time intervals, and in Fig. 4B, only the pseudopodial regions of a cell have been reconstructed.

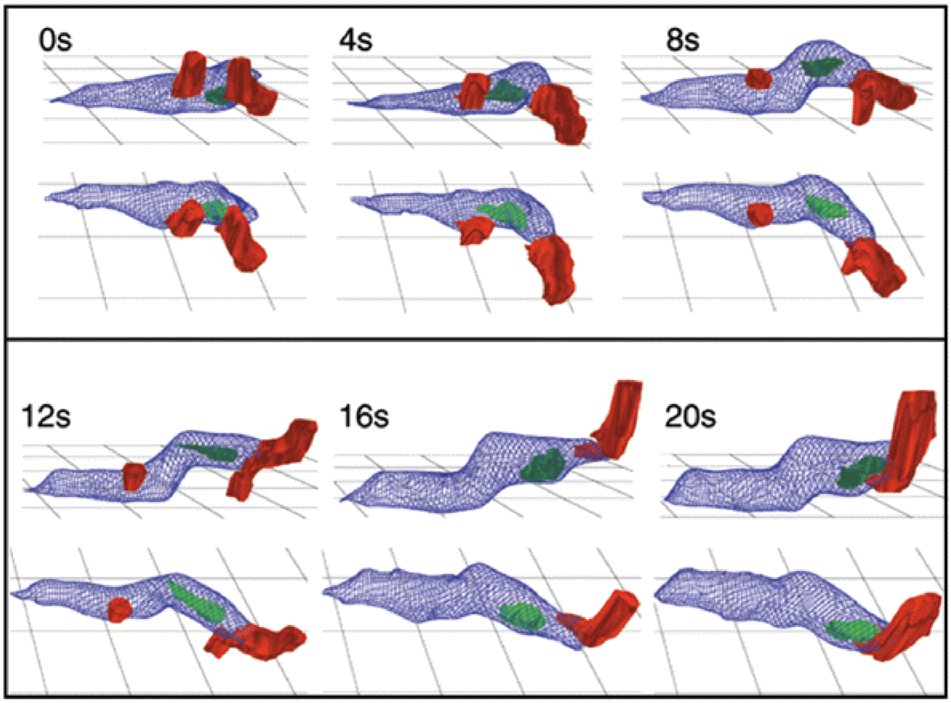

**FIGURE 3**. 3D-DIAS reconstructions of a wild type *Dictyostelium* amoeba crawling up a spatial gradient of the chemoattractant cAMP. The cell body is represented as a blue faceted transparent cage, the nucleus is represented as a nontransparent green image, and pseudopods are represented in a nontransparent red image. Time is in second(s). Reconstructions are viewed from two angles at each time point (see [12] for details).

## WHAT HAVE WE LEARNED ABOUT CELL BEHAVIOR USING 2D AND 3D MOTION ANALYSIS SYSTEMS?

Using both 2D-DIAS and 3D-DIAS, we have been able to develop a set of characteristics and rules governing wild type *Dictyostelium* cell behavior that provides us with the comparative

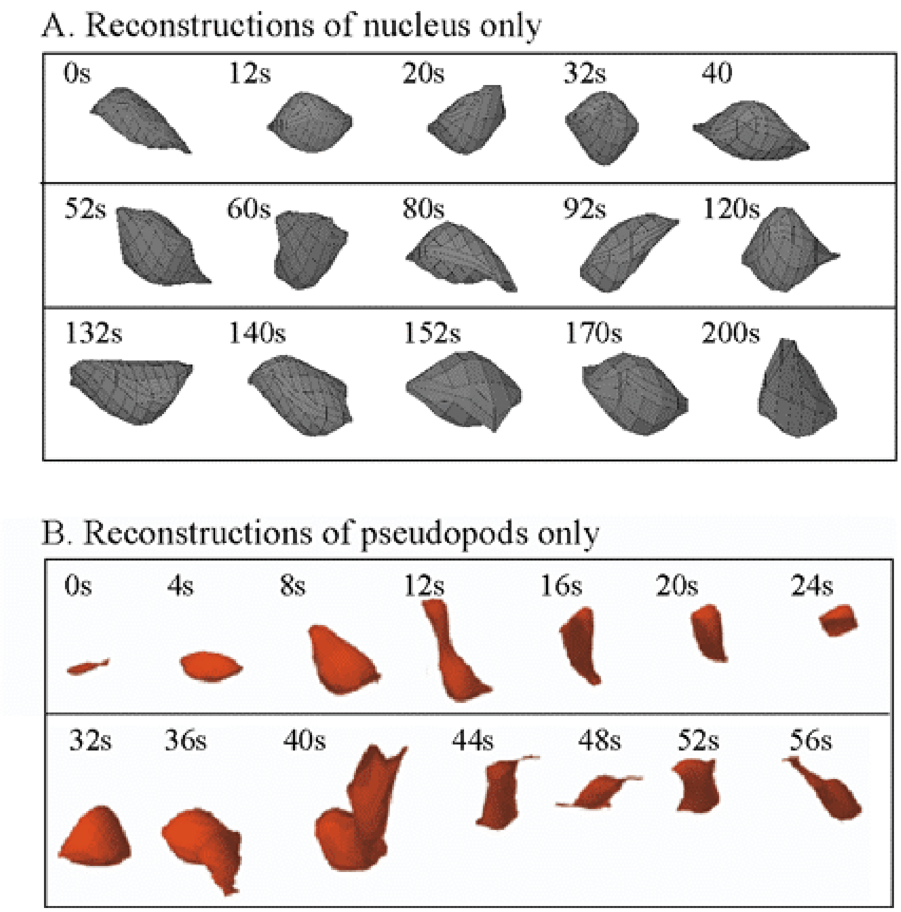

**FIGURE 4.** 3D-DIAS reconstruction of (A) the nucleus of a single cell over a 200-s time period and (B) the pseudopodial regions of a cell over a 56-s time period. The cell type is a *D. discoideum* amoeba.

context for assessing the role played by a particular gene in cell polarity, locomotion, and chemotaxis through mutant analysis. The sum of the characteristics and rules of cell behavior in buffer represents the "basic motile behavior" of a wild type cell. The basic motile behavior of a cell is then manipulated by the temporal, spatial, and concentration components of the natural relayed wave of cAMP to attain directed movement in the process of *Dictyostelium* chemotaxis. Although such a detailed list has only been developed for wild type *Dictyostelium* amoebae translocating in buffer, many of these rules and characteristics will no doubt prove common to other translocating animal cells, and are therefore worth considering in detail.

1. A cell is continually in the process of extending an anterior or lateral pseudopod[38].
2. A cell rarely extends more than one pseudopod at a time[38].
3. New pseudopods are preferentially extended from the anterior end of a cell[39,40].
4. The posterior end, or uropod, of a cell rarely is the location of new lateral pseudopod formation and represents the most constant component of a cell's morphology[36,39,40].
5. Anterior and lateral pseudopod formation are usually interspersed[38].
6. When extending a new pseudopod, the original pseudopod stops expanding, and if the new pseudopod assumes the role of a dominant anterior pseudopod, the original anterior pseudopod is retracted into the main cell body[38].

7. The position of pseudopod retraction is fixed in relation to the substratum, not in relation to the relative position along the cell axis[36].
8. Pseudopods form on and off the substratum. Pseudopods that first form on the substratum, or that first form off the substratum and then contact it, have a propensity to become the new anterior end of the cell, while pseudopods that form off and remain off the substratum are retracted[41].
9. Cells translocate in a cyclic fashion with a 1- to 2-min cycle most readily observed at a period between peaks in a velocity time plot. The velocity cycle includes phase I, in which the cell translocates along the substratum, and phase II, in which the anterior end of the cell extends upward in the z-axis[41].
10. It takes on average 25 s for a new pseudopod to grow to maximum size (15–20% of total cell area)[38].
11. A set of 2D and 3D motility and dynamic morphology parameters provide a quantitative description of the basic motile behavior of a wild type cell[31,36,42,43,44].

In *Dictyostelium*, these characteristics hold true for a variety of wild type strains, although some of the quantitative parameters, most notably cellular velocity, do vary. Hence, the behavior of each mutant must be compared to that of its parental strain. Many of these characteristics have also been established for other cell types. For instance, the 1- to 2-min velocity cycle has been demonstrated in human polymorphonuclear leukocytes[45], human CD4-positive T cells[19], human HIV-induced T cell syncytia[19], and human dendritic cells[46]. In *Dictyostelium*, this endogenous velocity cycle involves a 3D component[41], as diagrammed in Fig. 5. The cycle has been divided into two phases[38]. In phase I, which is associated with an increase in velocity, cells become elongate in the x,y-axis as they rapidly extend their new dominant anterior pseudopod along the supporting substratum (Fig. 5). In phase II, which is associated with a decrease in velocity, the anterior pseudopod lifts off the substratum and ascends in the z-axis (Fig. 5). In phases I and II, the nucleus tracks the dominant pseudopod, remaining positioned behind the interphase between the particulate cytoplasm of the cell body and the nonparticulate cytoplasm of the dominant pseudopod[12].

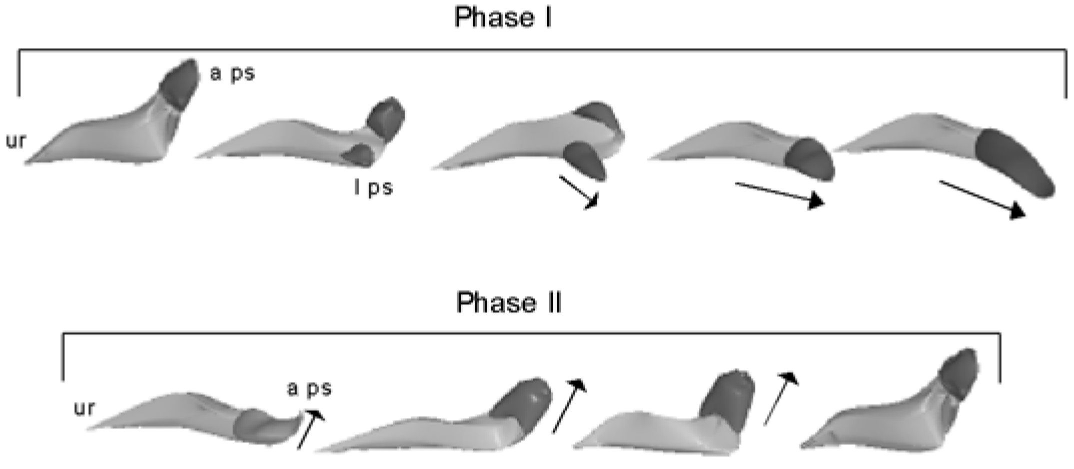

**FIGURE 5**. *Dictyostelium* amoebae crawl in buffer in a cyclic manner with a 1-min period ("behavior cycle"). This behavior cycle has two phases (I, II) depicted in 3D reconstructions. Pseudopods dark gray, cell body light gray. Arrow denotes direction of pseudopod extension (see [38] for details).

## EXAMPLES OF MUTANT ANALYSES THAT REQUIRED 3D-DIAS

Although the majority of 2D-DIAS analyses of mutant *Dictyostelium* behavior have revealed unique defects and have been instrumental in determining the role of mutated genes in motility and chemotaxis, there are examples in which the elucidation of mutant cell behavior required the additional information provided by 3D-DIAS analysis. The first example is the *Dictyostelium* null mutant of ponticulin, an actin-binding protein that associates with the plasma membrane[47]. 2D motion analyses of the deletion mutant of ponticulin revealed only a minor defect in the cell perimeter tracks[36]. The parameter tracks were slightly wider than those of control cells, suggesting aberrant lateral pseduopod behavior. However, all 2D parameters of motility and dynamic morphology were statistically indistinguishable from those of the parental strain. To pursue this minor aberration, a 3D analysis was performed. 3D reconstructions of living cells at short time intervals revealed that pseudopods that were formed by mutant cells off the substratum were not fixed in relation to the substratum as they were in wild type cells[36]. Instead, these pseudopods floated over the top of the cell towards the uropod as they were retracted. In wild type cells, homologous pseudopods remained fixed relative to the substratum as they were retracted. This aberrant behavior of pseudopods could not have been ascertained without 3D reconstruction and spatial demarcation of the pseudopods of live, crawling cells. The 3D analysis, therefore, revealed for the first time that ponticulin functioned to rivet the pseudopodial cytoskeleton to the general cytoskeleton of the main cell body, hence stabilizing its position in relation to the substratum[36].

An example of a mutant analysis that benefited from both 2D and 3D analysis was that of the myoA deletion mutant in *Dictyostelium*. MyoA is a member of the gene family of myosin Is[48]. 2D analysis revealed that *myoA* cells formed lateral pseudopods at roughly twice the frequency of wild type cells and turned twice as frequently[30]. Further analysis revealed that mutant cells broke wild type rules by extending multiple pseudopods at the same time[38]. A 3D comparison of pseudopods formed on and off the substratum revealed that the increase in the frequency of lateral pseudopod formation by *myoA* cells was restricted to those formed on the substratum[38]. Quantitative 3D analysis further revealed that mutant cells expanded a pseudopod on average at half the rate of a wild type cell, and mutant pseudopods took twice as long to attain full length as did wild type pseudopods[38]. 3D analysis, therefore, revealed for the first time that myosin A is involved in the selective suppression of pseudopods formed on the substratum[38]. These defects in the pseudopod dynamics of *myoA* cells are readily deduced from a visual comparison of 3D reconstructions of wild type cells (Fig. 3) and *myoA*$^-$ cells (Fig. 6).

A third example of a mutant analysis that benefited from 3D analysis is that of the deletion mutant of the protein kinase A regulatory subunit. Deletion of the regulatory subunit gene resulted in the mutant *pkaR*$^-$, in which PKA was constitutively active. 2D analysis revealed that *pkaR*$^-$ cells translocated at half the velocity of wild type cells, formed pseudopods at twice the frequency and were less elongate[49]. These defects persisted during chemotaxis in a spatial gradient of cAMP and caused a 50% decrease in chemotactic efficiency[49]. A 3D analysis revealed that *pkaR*$^-$ cells were ovoid instead of elongate, and formed complex pseudopods off the substratum, frequently at the top of the main cell body, rather than in the front of the cell along the substratum (Fig. 7). The 3D analysis revealed that PKA must remain deactivated in order for a cell to translocate efficiently in buffer, in a spatial gradient of cAMP and in the front of the wave, and left open the possibility that PKA activation played a role in the loss of cellular polarity at the peak of the natural chemotactic wave, a necessary component of chemotaxis in this organism. Other null mutants that have been demonstrated to be morphologically defective through 3D-DIAS analysis include *myoF*[50], the *myoA*/*myoR* double mutant[50], *Ddvasp*[51], *myoVii*[52], and *chc*[53].

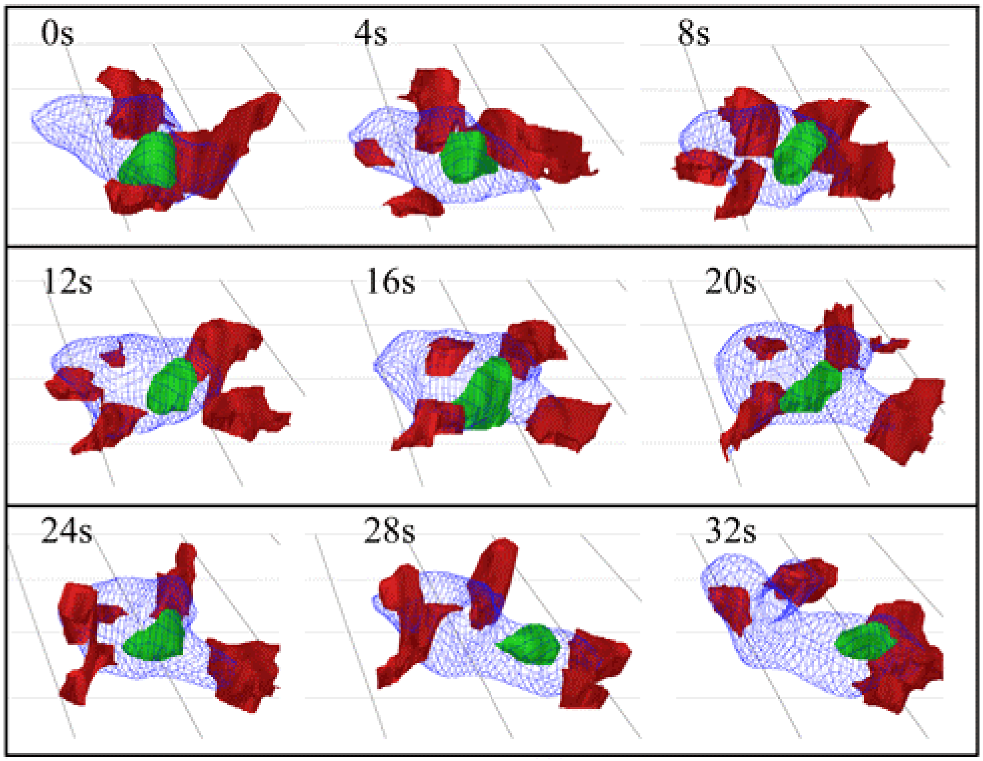

**FIGURE 6.** 3D-DIAS reconstructions of a *myoA*⁻ mutant *Dictyostelium* amoeba crawling in a spatial gradient of cAMP. Note multiple pseudopods, pseudopod placement, and loss of polarity. See legend to Fig. 2 for details of reconstructions.

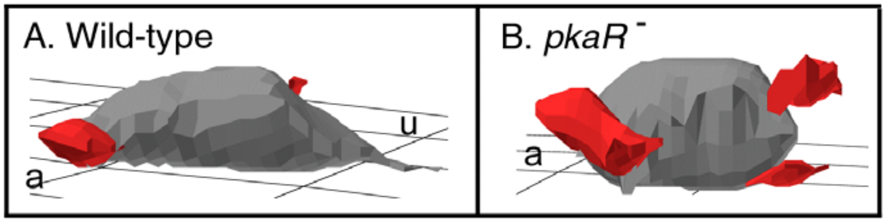

**FIGURE 7.** The major differences in cell shape between a wild type *Dictyostelium* amoeba and a *pkaR*⁻ cell in buffer is resolved by 3D-DIAS. Note the aberrant ovoid shape and formation of pseudopods off the substratum by *pkaR*⁻ cells.

## THE DEVELOPMENT OF 3D-DIASEMB

The strategy we have developed over the past 17 years did not end in single cell analyses. We realized that by increasing the number of individual trace slots in the 3D-DIAS program to 10,000 and adding new software for multicellular assembly, we could develop a new system, 3D-DIASemb, that would allow us to reconstruct in 3D every cell and nucleus through the early development of an embryo[54]. In this new system, 75 optical sections are collected in 2.5 s through DIC optics, and the process repeated every 5 s. The perimeters of each cell and nucleus are outlined in each optical section, the outlines are converted to β-spline representations, and the representations are used to generate transparent faceted images of each cell and nucleus in the developing embryo. Reconstructions from the 2- to 28-cell stage of a *Caenorhabditis elegans* embryo are presented in Fig. 8. In these reconstructions, mitotic nuclei are encapsulated and

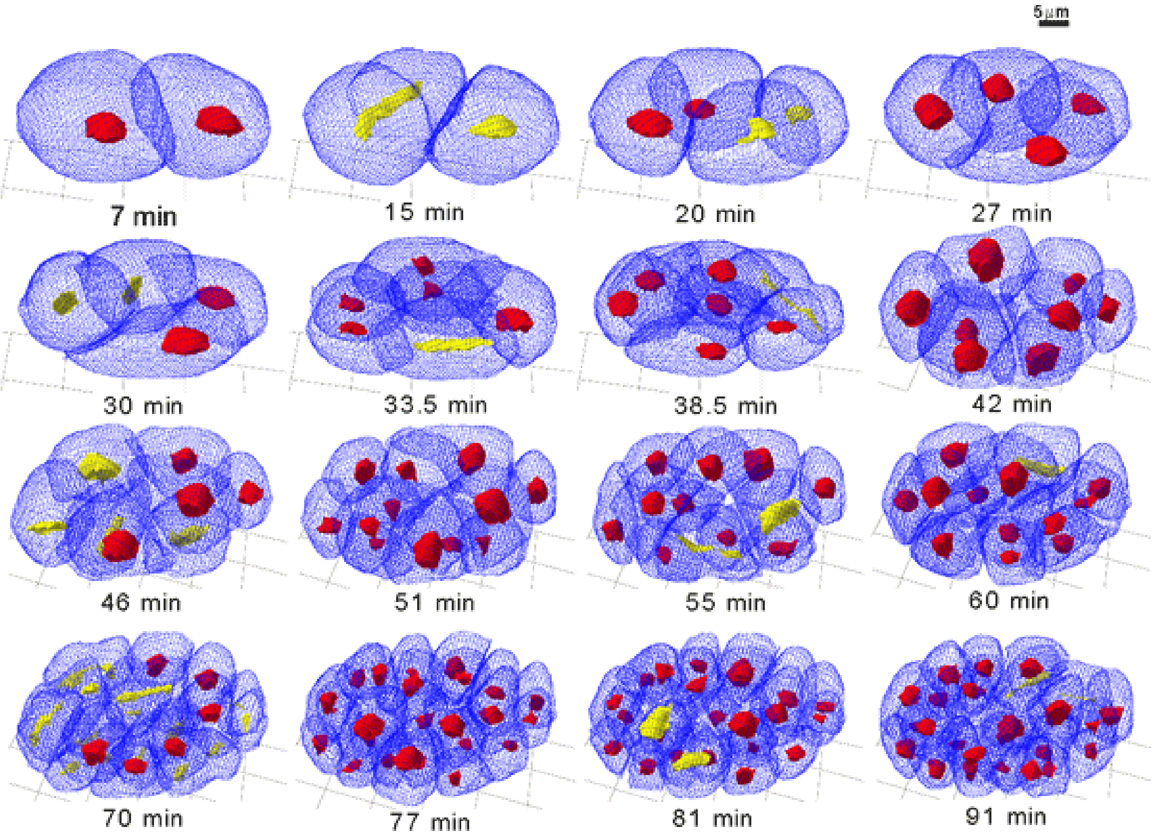

**FIGURE 8**. 3D-DIAS faceted reconstructions of the developing *C. elegans* embryo between 7 min (2 cell) ad 91 min (26 cell) of development. Cell surfaces are transparent blue faceted images, interphase nuclei red images and mitosing nuclei yellow images (see [54] for details).

color-coded yellow. Because the surface of each cell and of each nucleus is individually traced and then reassembled in space, one can reconstruct cell surfaces without nuclei or nuclei without cell surfaces (Fig. 9), or one can simply follow a single cell or nucleus in time and space (Fig. 10). In reconstructing nuclei, one can color code nuclear lineages (Fig. 9). In reconstructing single cell lineages, one can assess changes in single cell morphology. In the case of the "C" cell and its descendents in Fig. 10, a cell extension wraps partially around a neighboring cell, which then divides. This behavior was verified in a second reconstructed embryo[54]. It should be realized that the interval times between reconstructions in Figs. 8, 9, and 10 are several minutes. In fact, reconstructions can be made at intervals as short as 2.5 s. This provides an extraordinary level of resolution for motion analyzing small changes in cell shape and behavior, and cell-cell interactions in embryos and tissues.

Although the dynamic representations of a developing embryo are revealing, the real power of 3D-DIASemb ultimately will be in its capacity to compute more than 100 motility and dynamic morphology parameters for every cell and nucleus in a developing embryo[54]. More importantly, software now under development will provide new parameters that quantitate cell-cell relationships in space and time. The power of this system is obvious for a system like *C. elegans*. Just as in the case of *Dictyostelium*, high-resolution 4D phenotypes of mutant embryos will no doubt reveal new functions for a variety of genes that, through qualitative analysis, would have been interpreted as having no role in early embryogenesis.

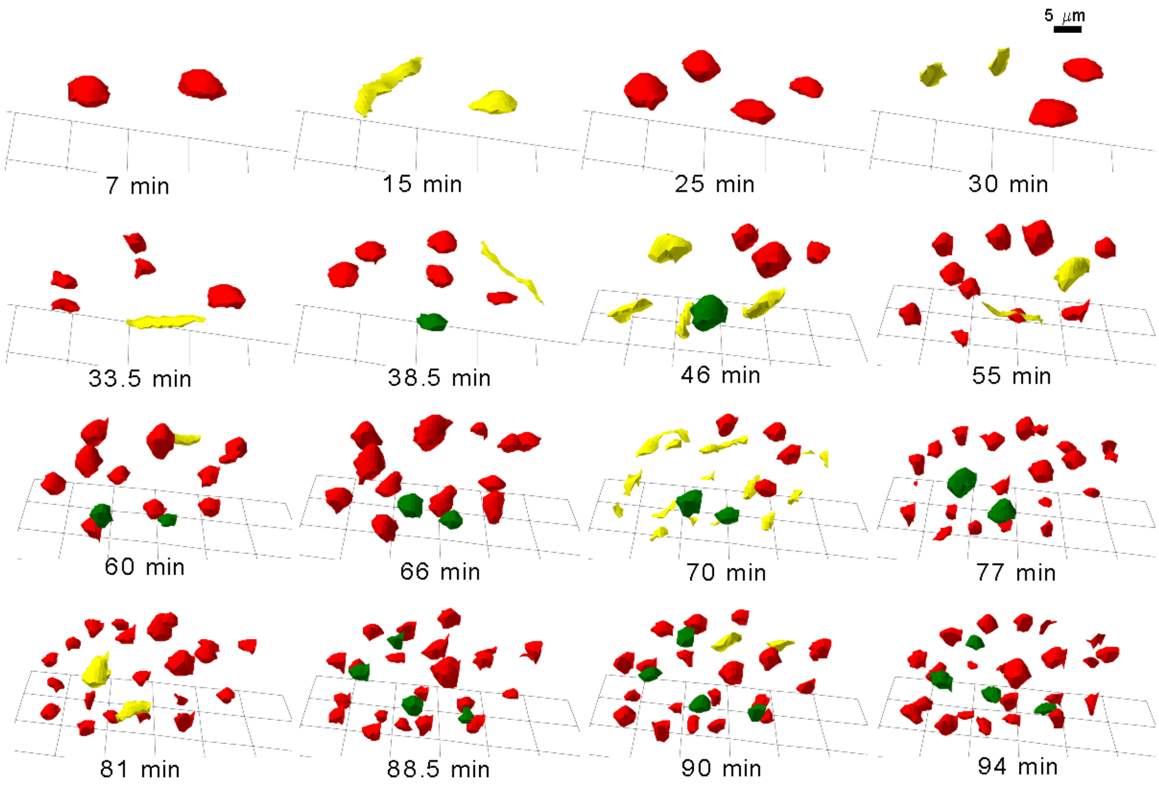

**FIGURE 9**. 3D-DIASemb provides reconstructions of nuclei during *C. elegans* embryogenesis. Interphase nuclei are color-coded red and mitosing nuclei yellow. In this sequence MS-derived nuclei are color-coded green. When undergoing mitosis, MS nuclei are also color-coded yellow (see [54] for details).

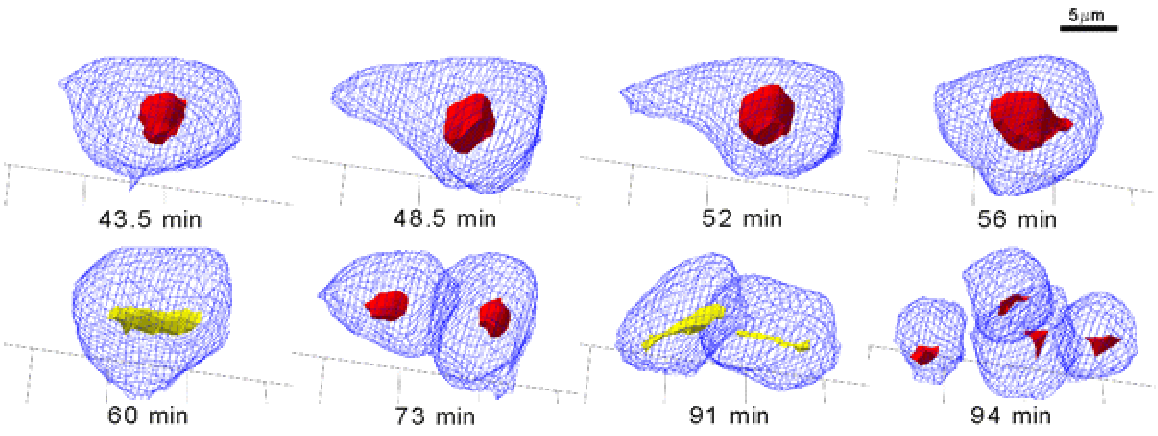

**FIGURE 10.** 3D-DIASemb allows presentation of single cell lineages, in this case the C-cell lineage between 43.5 and 94 min. Note the cellular extension between 48.5 and 52 min. Interphase nuclei are color-coded red and mitosing nuclei yellow (see [54] for details).

## CONCLUDING REMARKS

The capacity to reconstruct at short time intervals and then motion analyze live, crawling cells represents an underutilized technology with obvious potential for delving into the mechanisms underlying a variety of dynamic biological processes. As scientists delve deeper into the mechanisms of motility, chemotaxis, and morphogenesis, it becomes increasingly evident that cellular and subcellular behaviors are complex and involve unexpectedly large numbers of regulatory and cytoskeletal molecules that are involved in fine-tuning behavior. 2D-DIAS and 3D-DIAS provide the resolution necessary to define what aspect of the complex behavior of a cell is affected by a mutation, by a drug, by an extracellular chemotaxis signal, by an environmental perterbation, by the transition to metastasis, by a developmental program, or by interaction with another cell. 3D-DIASemb transfers this basic technology to the more complex embryo. Recently, the use of scatter labeling and two-photon laser scanning confocal microscopy has provided a new preparation to which 3D-DIAS can be used to reconstruct and motion analyze single cells deep in tissue[55]. DIAS-generated dynamic reconstructions can be viewed and downloaded from the W.M. Keck Dynamic Image Analysis Facility web site (http://keck.biology.uiowa.edu/).

## ACKNOWLEDGMENTS

Development and application of 3D-DIAS and 3D-DIASemb was supported by National Institutes of Health grant HD-18577 and by a grant from the W.M. Keck Foundation, both to D.R.S. P.H. was supported by a postdoctoral fellowship provided by the American Cancer Society [Grant PF-01-110-01-CSM].
